# Humans and Insects Decide in Similar Ways

**DOI:** 10.1371/journal.pone.0014251

**Published:** 2010-12-08

**Authors:** Philippe Louâpre, Jacques J. M. van Alphen, Jean-Sébastien Pierre

**Affiliations:** CNRS UMR6553 EcoBio, IFR90/FR2116 CAREN, Université de Rennes I, Campus de Beaulieu, Rennes, France; University of Bristol, United Kingdom

## Abstract

Behavioral ecologists assume that animals use a motivational mechanism for decisions such as action selection and time allocation, allowing the maximization of their fitness. They consider both the proximate and ultimate causes of behavior in order to understand this type of decision-making in animals. Experimental psychologists and neuroeconomists also study how agents make decisions but they consider the proximate causes of the behavior. In the case of patch-leaving, motivation-based decision-making remains simple speculation. In contrast to other animals, human beings can assess and evaluate their own motivation by an introspection process. It is then possible to study the declared motivation of humans during decision-making and discuss the mechanism used as well as its evolutionary significance. In this study, we combine both the proximate and ultimate causes of behavior for a better understanding of the human decision-making process. We show for the first time ever that human subjects use a motivational mechanism similar to small insects such as parasitoids [1] and bumblebees [2] to decide when to leave a patch. This result is relevant for behavioral ecologists as it supports the biological realism of this mechanism. Humans seem to use a motivational mechanism of decision making known to be adaptive to a heterogeneously distributed resource. As hypothesized by Hutchinson *et al.* [3] and Wilke and Todd [4], our results are consistent with the evolutionary shaping of decision making because hominoids were hunters and gatherers on food patches for more than two million years. We discuss the plausibility of a neural basis for the motivation mechanism highlighted here, bridging the gap between behavioral ecology and neuroeconomy. Thus, both the motivational mechanism observed here and the neuroeconomy findings are most likely adaptations that were selected for during ancestral times.

## Introduction

From basic behaviors to complicated decisions, all animals, including humans, have to make choices throughout their life in order to maximize their utility function [5]–[8]. The choice of the best option can be defined either in proximate terms (satisfaction, welfare, reinforcement) or in ultimate functions (fitness); however, proximate decision cues are supposed to have a predictive value for fitness. Neuroeconomists study the proximate mechanisms of such decisions in humans and look at the role of the different brain areas in the decision process [9]. On the other hand, behavioral ecologists interpret the proximate mechanisms of decision-making in animals within the framework of a natural selection process [10]. We note that similar problems are studied in both fields, but from a different point of view. For example, human and animal decisions in terms of foraging activities are studied in situations where the resource distribution is clumped in patches (e.g. information on the internet for humans [11] and prey for animals [12]). Thus, it is important to decide when the current action should be continued (foraging on the current patch) and when to switch to another action (leave the patch) in order to maximize the yield. This problem originated from Charnov's well-known Marginal Value Theorem [12]. He identified the optimal decision to leave the current patch as a function of the rate of energy gain in the environment. Iwasa *et al.* [13] later showed that the optimal decision should rely on a Bayesian estimation of the number of prey remaining in a patch.

**Figure 1:**
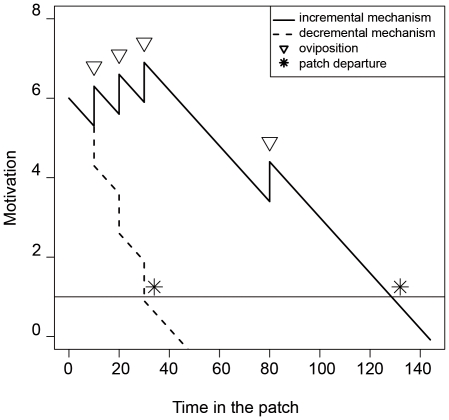
The motivational mechanisms of decision-making in parasitoids and the bumblebee. The insect enters a patch with an initial motivation to stay, which decreases linearly when no reward is found (in the case of parasitoids, hosts for laying eggs). Note that the motivation suddenly increases (incremental mechanism —) or decreases (decremental mechanism – –) when a reward is found (Δ). The decision to leave the patch occurs when the motivation falls below a given threshold (*). From Waage (1979).

Behavioral ecologists have suggested that insects such as parasitoids and bumblebees use a motivational mechanism [1], [14] (Figure 1) to perform these tasks. Here, the term motivation is defined according to the implicit motivational system described by McClelland *et al.* [15]: motivation is a biological variable that drives a behavior in the sense that it energizes, directs and selects behavior [16], [17]. Dorman and Gaudiano [18] following Hull [19] and Skinner [20], provide a very similar definition: “the internal force that produces actions on the basis of the momentary balance between our needs and the demands of our environment”. These definitions apply to both humans and animals, and this concept is assigned to the category of hidden or latent variables, which cannot be measured directly but only by its correlation with an observable behavior. According to the motivational hypothesis of making the decision to leave the patch, an animal enters a patch with an initial motivation that decreases monotonically as long as no rewarding item is found. Each time an item is discovered, the motivation suddenly increases (positive reward, incremental model) or decreases (negative reward, decremental model). The animal leaves the patch when the motivation falls below a certain threshold. This process was first described in Waage's model [1], in which the value of the increment depends on the time since the last capture.

We examine here whether human subjects follow a similar process in a foraging task. In order to simplify the motivational model, we assume here that the value of the increment constant does not depend on the time since the last discovery. We will further examine whether or not this simplification holds. Under this assumption, the level of motivation *m*(*t*) at time *t* in the current patch is simplified as:
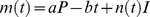
(1)where *aP* is the initial tendency to stay in the patch, *b* the slope of the linear decrease, *n*(*t*) the number of hosts met by time *t* and *I* the value of the motivation increment (greater than 0) or decrement (less than 0). Otherwise, if we accept Waage's original model, the course of motivation should be written as:
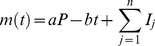
(2)


The rule emerging from these processes is quite simple: the animal leaves the patch when m(*t*) falls under a given value m_0_ which can be fixed at 0 without the loss of generality. The sign of *I* is important for the adaptive value of the behavior. It is now accepted that the adaptive value of the sign of *I* depends on the distribution of the prey or hosts among the patches. This concept arose from the work of Iwasa *et al*. [13]. They devised a process that is quite different from Waage's model but which share some of the same features. They addressed the question of how a Bayesian forager can estimate the number of items remaining in a patch when it has caught *n* of them after having spent a time *t* in it. They first showed that for an over-dispersed distribution, the pair (*n*, *t*) is a sufficient statistic for that. Secondly, they showed that each discovery and consumption of a new item resulted in a sudden increase of this estimation, such as the negative binomial, whereas they resulted in a sudden decrease for an underdispersed distribution such as the binomial. As the Marginal Value Theorem is based on a rate of discovery and depends on the number of remaining items in the patch, many authors have concluded that the incremental case for Waage-like models was adaptive in very clumped distributions and that the decremental one was adaptive for even distributions (see [21] for a review).

Behavioral ecologists have hypothesized that natural selection tailored decision-making based on a motivational process, which is adapted to the resource distribution that animals experience [13]. As mentioned above, it is not possible to record the motivation itself, only proxies. Human beings are the only animals that can be asked to assess and communicate their own motivation. Verbal self evaluation by the subjects may be a better proxy than the behavioral ones available in animals. Psychologists admit that humans are able to accurately report some cognitive processes by introspection [22], [23]. For example, Corallo *et al.* [24] demonstrated the remarkable accuracy of introspective estimates of task duration. Their results show that subjects excel at estimating the duration of their internal process. With respect to motivation, different self-reported measures are routinely used to assess the motivational state of humans and the psychometric properties of these measures have been widely supported [25]. There are also many psychological and neuronal evidences of a human introspective system providing a subjective image of an emotional state [26]. In this sense, humans appear to be an appropriate model to study this motivation-based mechanism. The method, however, is far from being unbiased and its results must be discussed thoroughly.

Many authors have studied how such a decision could be mediated in the human brain in relation to the predictability of finding a resource [27]–[30]. Quite recently, Hutchinson *et al.* [3] and Wilke *et al.* [31], using two different electronic games, found the fundamental result that human decision-making is insensitive to the resource distribution. In this article, we primarily address the question of the likelihood of a motivational process sharing the features proposed by Waage and other authors, in the case of human subjects faced with a foraging task. In particular, we ask if it is possible to find evidence of the increase and decrease of motivation linked to the amount of time spent without finding any items (weariness), the discovery of items (reinforcement) or the finding of an empty chest (disappointment). We will also examine if this process is sensitive to the distribution of items among patches during a time-limited foraging task.

For this purpose, we devised a foraging computer game and asked the subjects to evaluate their own motivation during the task. We recorded the foraging behavior and declared motivational states of the subjects in various environments differing in terms of the resource distributions among patches. The resource was either evenly distributed or aggregated (low *vs*. high levels of variance, respectively).

## Materials and Methods

**Figure 2:**
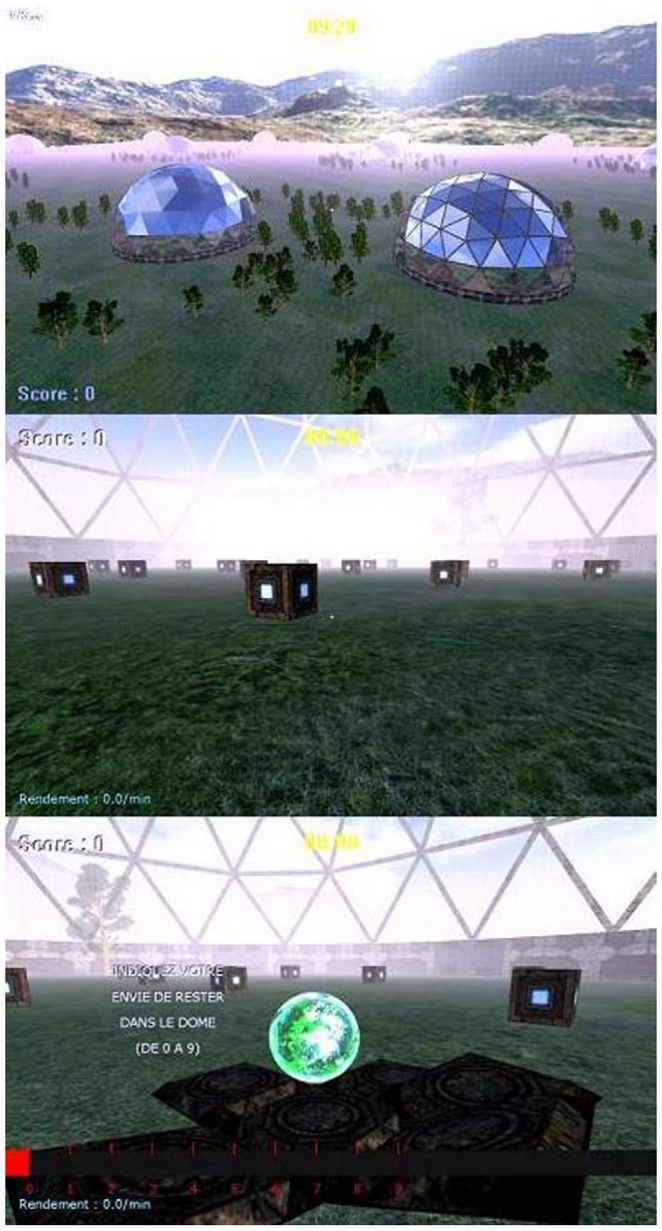
Screenshots of the virtual foraging game. Top: An overview of the virtual meadow with the spatial distribution of the patches. Middle: The chests when the player enters a patch. Bottom: The player opened a filled chest and found the resource. The French text on the bottom screenshot asks the subject to note his motivation to stay in the dome (from 0 to 9). At any given time, the player knows both the number of items found (left top corner of the screen) and the instantaneous yield (left bottom corner).

We developed a software system in the style of a FPS game (“first person shooter”, a video game centering the player inside a realistic 3D-environment) that records the instant-by-instant actions of individuals foraging for patchily distributed resources during a period of 30 min. The potential locations where resource might be found are represented by chests. Each chest either contains the resource (depicted by a little green sphere, see Figure 2) or it is empty. 49 Chests are distributed over 40 patches (in the shape of a dome) that are randomly distributed in a meadow. To disorientate the players inside the domes, each dome has 8 doors at its periphery and the chests are randomly distributed inside the dome area. The meadow size is 3600 virtual distance units in width and 3600 in height. The player speed is approximately 16 units×min^−1^. A fog reduces the visibility to prevent the subjects from visually spotting the distribution of the domes or to glean any information about the quality of the dome. Therefore, the players can only gather information by active foraging. Once inside a dome, the game is interrupted each time a chest is opened. In order to continue foraging in the patch, player must evaluate his motivational level typing in a number between 0 and 9 on the keypad, where 0 means “I want to leave the current dome and forage elsewhere” and 9 means “I want to continue to forage in the current dome”. After being opened, each chest appears as it did before but no longer contains a sphere inside. With this procedure, the total number of chests inside a dome is constant and the player cannot distinguish an already opened chest from one that has never been opened. Each dome can be exploited only once. After leaving a dome, its colour changes to red, indicating that it has already been exploited, and all of the doors are closed. During the experiment, the program records all of the player's actions and the declared motivation level in real time. To test if humans adapt their behavior to the resource distribution, five map types are defined. In each map, the spatial distribution of the domes throughout the meadow is the same; however, the domes differ in terms of their content. The ratio of filled/empty chests is based on three probability distributions: the number of items per patch is either Poisson distributed (random distribution, 1 map, *λ* = 30), binomially distributed (even distribution, 2 maps, respectively. *p* = 0.25 and *p* = 0.4) or negative binomially distributed (very clumped distribution, 2 maps, respectively. *k* = 0.5; *µ* = 10 and *k* = 0.1; *µ* = 15). Based on the work of Iwasa *et al.* [13], we assume that the optimal theoretical strategy differs according to the resource distribution.

Ninety-two subjects living within the vicinity of the University of Rennes 1 were recruited (53 men, 39 women; aged between 18–55 and 18–57, SE 8.01 and 10.50) for the software beta-test (12 subjects) and for the experiment (80 subjects). Each subject played the game only once. All of the subjects were volunteers and did not receive any payment. The best five scores of the five maps were published at the university. Because the recruitment was passive, we considered the subjects to be motivated to win the game. One of the five resource distributions is randomly assigned to the subject without any indication. Following Hutchinson *et al.* [3], the goal and method are explained with a slideshow on a computer during a period of 5 min. The 30 min experiment is preceded by a 4 min practice session with the same resource distribution. This familiarizes the player with the game, keyboard manipulation and measurement of his motivation. According to learning theories, the subjects need some experience within an environment in order to stabilize their foraging strategy. For this reason, we only considered the last half of the visited domes in each player's record. We first fitted the motivational model to the player's own assessment of his motivation course using by five linear and non-linear methods.

### Direct linear fitting (lm function of R)

Equation (2) can be fitted by a multiple linear regression with *t*, the time spent in the patch and *n* the number of items found at time *t* as an independent variable. This is model 1. An alternative is to consider whether the opening of an empty chest has a decremental effect. This is model 2, and the equation becomes:
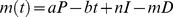
(3)where *m* is the number of empty chests opened at time *t* and *D* is their decremental effect. This first approach has some inconveniences. The estimate is unconstrained and may be negative or greater than 9. Testing its significance relies on the classical hypothesis on the normality, homoscedasticity and independence of the residuals which are dubious when the observed variable is both discrete and bounded.

### Non-linear fitting (*nls* function of *R*)

If the bounding of the observed motivation is taken into account, it can be introduced as a “chop” function leading to the following model:
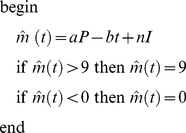
(4)


This is model 3. Its inconvenience is to increase the kurtosis of the residuals distribution by forcibly setting some of the residuals to zero.

### Generalized linear models (*glm* function of *R*)

The last approach considers the declared motivation as a binomial variable taking discrete values from 0 to 9, linearly linked to the external variables *t, n* through a logit link. The advantage is to avoid the hazardous hypothesis of the normality and independence of the residuals. The inconvenience is that the constancy of the residuals is no longer valid on the measurement scale, but only on the logit scale. On the scale of the measurements, the increments are large when the declared motivation is close to 4.5 and small when it is close to either 0 or 9. However, this might be realistic. The model 4 is then:
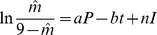
(5)where 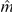 is the estimate of the declared motivation. Model 5 is the same as model 4 but it incorporated the effect of opening empty chests.

These five sorts of models correspond to three different psychological hypotheses:

- Linear approach (models 1 and 2)
  - The declared motivation is a faithful reflection of the motivational state of the individual, this motivational state is bounded and the subject has no difficulty to map it on the obliged interval.
- Non-linear approach (model 3)
  - The real motivation is not bounded, the subject has to chop it and a declared motivation of 9 means “9 or more” just as 0 means “0 or even less”. However, the real motivation is a linear function of *t* and *n*, and the interval [0,9] is a window to it.
- Generalized linear model approach (models 4 and 5)
  - The real motivation is not bounded, the subject has to map it on the interval [0,9] and the mapping is of logit type. This means that it requires more increments to pass from 8 to 9 than from 4 to 5.

It was therefore interesting to see globally which of these models fit best overall. To assess the significance of the fit, we used the Bonferroni procedure at the level of risk 0.05/80. A significance level such as this must be regarded carefully because of the unavoidable correlation between residuals in time series. In our case, this level only indicates if the fit of the model is better than the null model. We then focused on (i) rho2, which is a statistical indication about how well the model fits the observed motivation and (ii) the AIC (Akaike's information criterion), which measures the goodness of fit of the model. We point out the fact that one of the variables included in models 2 and 5, the number of empty chests opened, is directly linked to the time spent on the patch because it takes time to open chest and the number of empty chests opened is proportional to the time needed to open them. However, the AIC is known to detect strongly correlated variables and the addition of a strongly correlated variable could increase the AIC, thus indicating that the variable adds nothing to the model. Moreover, the linear model can solve the partial confusion between the variables (such as time and the number of empty chests opened). These five models were fitted individually to each participant. In the end, we kept model 5, as explained in the “Results” section. After examining the variety of the subjects' reactions, we discovered that it would be very complicated to analyze the data if the subjects were considered as a random factor in an overall analysis, especially since it is important to visually examine the fit of each individual run with the model.

We then used Cox's proportional hazard model to determine the effect of the different stimuli on the tendency to leave the patch. This model allows us to estimate the hazard rate at time *t*, which can be interpreted as a tendency to leave the patch. We estimated the effect of the different intra-patch cues (opening a filled chest, opening an empty chest), extra-patch cues (total number of empty/filled chests before entering the patch, travel time between two successive patches) and fixed covariates (sex, age, laterality, knowledge of the optimal foraging theory) on the tendency to leave the current patch. We integrated a random effect modelized by the Gamma frailty model describing the excess risk above any measured covariates due to multi-censored data for each individual [32]. The idea is that individuals have different frailties and those individuals who are more frail will leave the patch earlier than the others. The hazard rate *h_j_*(*t*) of the *j* ^th^ individual at time *t* in the patch is given by:
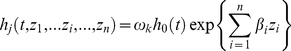
(6)where *h*_0_(*t*) is the baseline hazard function to leave the patch depending only on the time spent on it (all of the covariates are set to zero) and *z_i_* are the covariates which influence the tendency to leave the patch with *β_i_* contributions. ω_k_ is the frailty parameter for each subject. The patch-leaving tendency is reduced if a hazard ratio (exp{∑*β*_i_ *z*_i_}) is lower than 1, whereas a hazard ratio greater than 1 increases this tendency. Finally we investigated the plausible relationship between the results from model 5 and Cox's model: we took into account the simplified Waage's model which explained more than 50% of the variance (*R^2^*≥0.5). According to Pierre [33], Waage's parameters cannot be identified separately on the basis of the mere observation of the patch residence time. Only *aP*/*b* and *I*/*b* can be identified separately. We used another Cox proportional hazard model that integrates only the effect of opening a filled chest on the leaving tendency for each individual. We then correlated the value of the covariate *β* corresponding to the effect of encountering an item on the patch-leaving tendency and the measure of an associative factor from Waage's model fitting.

All of the computations were done with the R 2.10.0. software (R development core team, 2009).

## Results

During the 30 min experimental session, a player visited an average of 9.6 domes (SE 0.36), opened 252 chests (SE 7.63) and found 65 spheres (SE 3.08). The mean residence time in a dome was 758s (SE 2.33). The mean gain rate at the end of the game was 3.46 spheres.min^−1^ (SE 0.10, range 0–18.2 spheres.min^−1^).

Of the 80 motivational trajectories (one per subject), 78 showed a significant fit to model 5, the Generalized Linear Model with covariates *t*, *n* and *nloose* (see “Materials and Methods” section for a description of all of the models) at the Bonferroni level *α*' = 0.000625 (0.05/80, *χ* ^2^). However, this significant level only indicates that our model fits the data better than the null model. According to the AIC criterion, this model gives the best results in 34 out of 80 cases (Table 1). The simple unconstrained linear models are the best ones in only a few cases. The non-linear chopped model and the generalized linear model 4 appear to be equivalent. In more than 42% of the cases, the inclusion the number of empty chests opened decreases the AIC of the model. This demonstrates the decremental effect of opening empty chests even if the AIC gain is low in all of the cases.

**Table 1:** Number and percent of cases where each model and model class appears to be the best using the AIC criterion.

Model number	Best model	%	Model class	Best class	%
1	3	3.75	Linear	7	8.75
2	4	5			
3	19	23.75	Non linear	19	23.75
4	20	25	Generalized linear	54	67.5
5	34	42.5			

**Figure 3:**
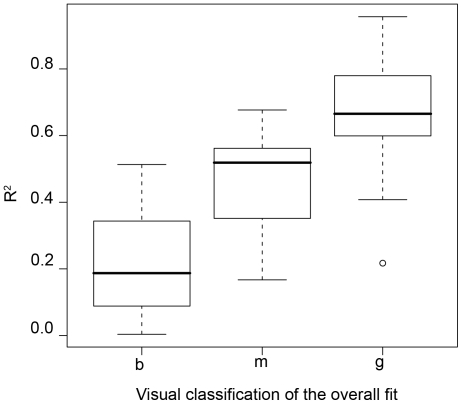
Relationship between the visual classification of the overall fit and goodness of fit of individual motivation, given by *R* ^2^ in the adjusted model 5. b: bad visual adjustment (strong discrepancy between the course of motivation and the fitted values); m: medium visual adjustment (partial consistency); g: good visual adjustment (total consistency, no systematic bias).

Bellow, we will refer to model 5 as the best fitting model. Referring to the hypothesis that we formulated in the “Material and Methods” section, this indicates that subjects are well able to map the evaluation of their motivation into the 9-point scale that we specified. Taking in consideration the effect of opening an empty chest appears also to be important. However, the overall fit of model 5 does not represent the quality of fit, which is very different from one individual to another. After an examination of the visual fit of each model line to each motivation course and other criteria such as the presence of visible bias on the residuals *vs.* fitted values diagrams, we then decided to classify the results into three categories: (i) G - good. The line of the fitted values plotted against time correctly follows the line of the motivation declared by the subjects. No systematic bias is observable on the graph of the residuals against fitted values. (ii) M - medium or acceptable. The line of the fitted values correctly follows at least some parts of the graph, generally in some patches. (iii) B - bad or poor. The line of the fitted values is not very coherent with the course of the motivation, strong biases are observed in the graph of the residuals. We then compared this visual appreciation to the determination coefficient of the model as a measure of the goodness of fit. Figure 3 shows the relationship between this visual and partly subjective classification and *R* ^2^. Clearly, there is a strong relationship between the classification and goodness of fit. We found this procedure better than a cut on the basis of the *R* ^2^ because as shown in this figure, a high *R* ^2^ value can mask strong biases. On this basis, 49 fits were considered as good (61.25%), 19 as medium (23.75%), and 12 as bad (15%). If the good and medium cases are added up, there are 68 acceptable fits and 12 bad (unacceptable) fits. We concluded that 85% of the subjects show a course of their declared motivation which is coherent with a process in which the discovery of an item in a chest has an increasing effect and the opening of an empty chest a decreasing effect.

**Figure 4:**
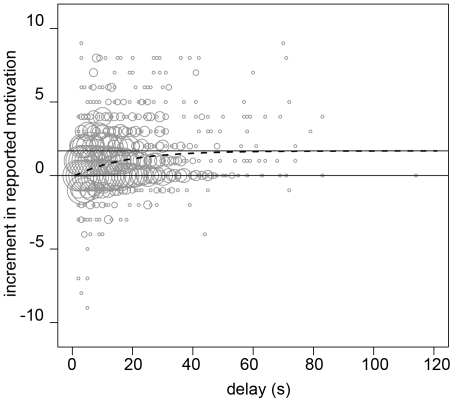
Relationship between the size of the increments and the delay since the last discovery of an item. Each petal of the sunflower plot represents one overlapped point. The relationship (red dotted line, *increment = 13.67627*(1-exp(-0.06025*delay*))) is in accordance with Waage's model but the effect is weak and blurred by a large noise.

We also verified Waage's hypothesis linking the size of the increments in reported motivation to the delay since the last discovery of an item. We actually found a weak effect from this delay (Figure 4). The shape is coherent with Waage's idea of an increase from zero to a maximum value, and for this reason it fitted the model:
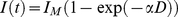
(7)by non-linear least squares (*nls* function of *R* base), where *D* is the delay since the last discovery, *α* a parameter and *I_M_* the maximum possible value of the increment. Although significant, the model only explains 6% of the variance (*R* ^2^ = 0.06) and Figure 4 shows how the variables are the observed values and the poor contribution of the model. We conclude that there is something true in Waage's intuition in the case of man, but that this effect is blurred by a large noise.

**Figure 5:**
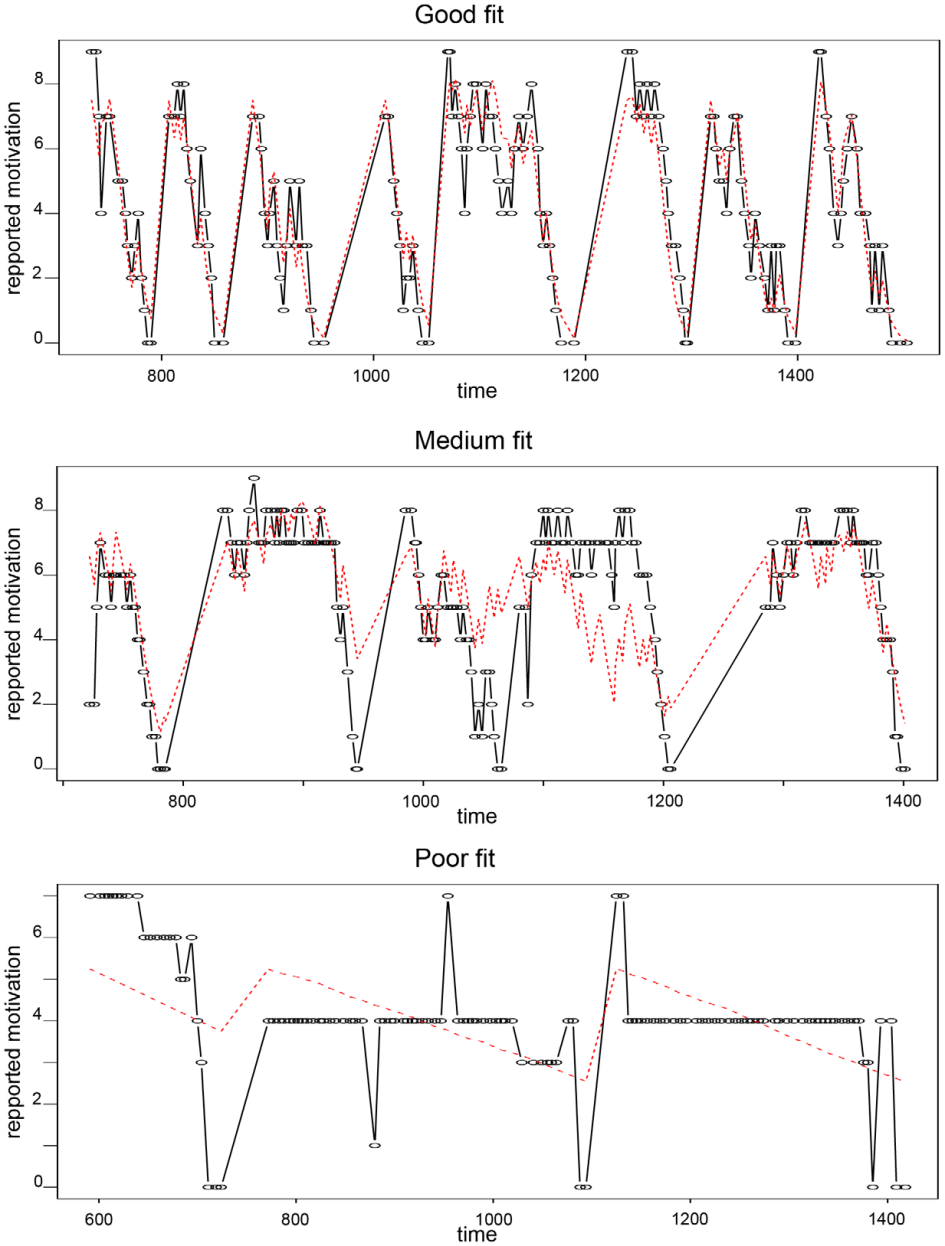
Motivational course during the foraging time of a player classified as “poor”, “medium” and “good”, using the motivation adjustment described in the text.

Figure 5 shows the fit of each of the three classes of the model 5 in one individual (good, medium and poor fit, respectively). A feature that is worth noting is that the fit classified as “bad” is completely different from the others. It is clear that these subjects do not exhibit, at all, a course of their evaluated motivation that is compatible with Waage's model. We were not able to determine if it resulted from an inability to assess their own motivation or if it is due to a completely different strategy. We discarded these individuals from further comparisons between Waage's process and the analysis of incremental/decremental effect by Cox, given that they did not follow Waage's process.

For all of the players, the estimated values of the motivational model 5 (initial motivation, decrease of motivation, motivational increment and decrement due to the opening of an empty chest) did not differ significantly in different environments (respectively. *F* = 0.13, *df* = 4; 71, *NS*, *F* = 1.01, *df* = 4; 71, *NS*, *F* = 0.66, *df* = 4; 71, *NS and F = 1.93, df* = 4,71, *NS*). The players entered a patch with a high motivational level (initial motivation  = 7.49, after inverse logistic transform, SE 1.73) that decreased over time (b = 0.062s^−1^, SE 0.00505 on the logit scale) and left the patch when the motivational level approached 0. Opening a filled chest increased the motivational level by 0.47 (SE 0.53) units on average (on the logit scale), in every resource distribution scenario. The model was therefore incremental, respective to the discovery of a filled chest in every environment. We should note that on the motivational scale, neither the increments *I*, nor the slope of decrease *b* are constant. They are curved functions depending on *t* and *n*.

The analysis of the residence time by Cox's proportional hazard model [34]–[36] (see the “Materials and Methods” section) supported our previous results: opening a filled chest decreased this tendency regardless of the resource distribution (Table 2; the effect of the resource distribution – not shown – was not significant). Opening an empty chest increased the player's tendency to leave the patch but this effect is lower than the incremental effect of finding a resource. In order to compare this with an alternative model to explain the patch leaving decision, we added another covariable independent of the number of discoveries but dependent of time since the last discovery (giving-up time). As shown by Hutchinson *et al.* [3], the more time that has passed since the last discovery, the lower the tendency to leave the patch. The effect of the number of discoveries influenced the leaving-tendency 16 times more than the giving-up time. The extra-patch experience also changed the tendency to leave: if more filled chests were opened in the previous patches, the tendency to leave the visited patch was higher. Conversely, if the more empty chests were opened in the previous patches, the tendency to leave the visited patch was lower. In addition, when the travel time between two successive patches was longer, the tendency to leave the visited patch decreased. To find possible effects of the different categories of individuals, different factors and covariates were included in Cox's proportional hazard model such as age, sex, laterality, type of environment, knowledge of Optimal Foraging Theory, and familiarity with video games. Only two fixed covariates influenced the tendency to leave: age and laterality (Table 2). Being older or left-handed decreased the tendency to leave a patch when compared respectively to being young or right-handed.

**Table 2:** Estimated regression coefficients (*β*), standard errors (SE) and hazard ratios (exp (*β*)) for covariates that have a significant effect (*P*-value<0.05) on the patch-leaving tendency of humans in a multi-patch environment. *χ* ^2^ corresponds to the likelihood ratio test.

	*β*	exp(*β*)	SE(*β*)	*χ* ^2^(*df*)	*P*-value
**Effect of the within-patch experience**					
Number of full chests opened so far	−0.1631	0.85	0.0205	63.17	1.9e^−15^
Number of empty chests opened so far	0.0519	1.053	0.0172	9.13	2.5e^−03^
Time since the last capture	−0.0097	0.99	0.0026	13.90	1.9e^−04^
**Effect of the previous experience**					
Total number of filled chests opened before entering the patch	0.0188	1.019	0.0054	12.18	4.8^−04^
Total number of empty chest opened before entering the patch	−0.01	0.99	0.0023	17.85	2.4e^−05^
**Effect of fixed covariates**					
Age of the subject	−0.068	0.934	0.0181	14.14	1.7e^−04^
Laterality (left-handed)	−1.29	0.275	0.4136	9.73	1.8e^−03^

*Note: the overall significance of the fitting model: χ^2^ = 534, df = 67.4, P-value<0.001*.

**Figure 6:**
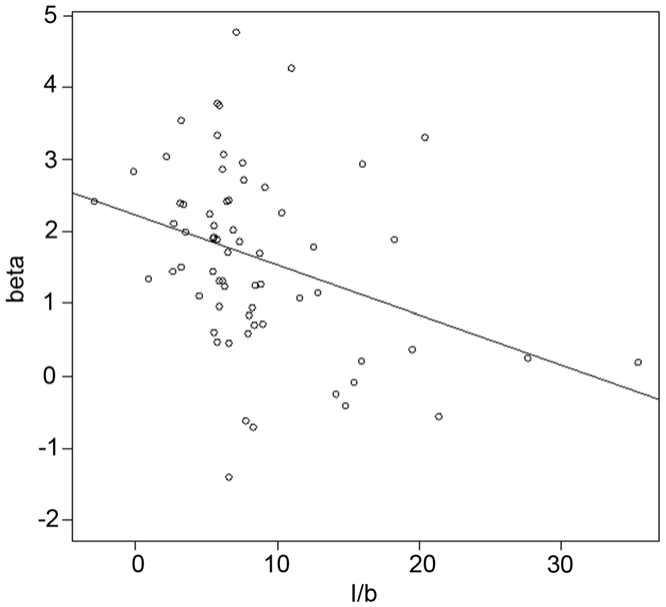
Relationship between Cox's model *β* and the term I/b from Waage's model. The Y-axis refers to the values of the effect of opening a filled chest plotted against on the hazard rate for Cox's proportional hazard model. The X-axis is obtained from the motivational model.

Figure 6 shows the correlation between the values of the covariate *β*, corresponding to the effect of encountering an item on the patch-leaving tendency, and the values of the term *I*/*b* calculated by fitting the motivational model (number 5, see the “Materials and Methods” section). We found a significant relationship between the fitted values of *I*/*b* in Waage's model and the value of Cox's model covariate (*R* ^2^ = 0.27; regression equation *beta* = 2.15-0.066*I*/*b*; *F.* test  = 17.57; *df = *1;64, *P-*value <0.001). The subjects that reported a larger increase in motivation to stay when a sphere was found (relative to the tendency of motivation to decrease over time) were those subjects who were not inclined to leave a patch when they found a sphere.

## Discussion

Our results provide evidence for a simple decision-making process, similar to what is generally assumed in parasitoids [37] and bumblebees [2]. Humans decide whether to stay in a patch or move to another one according to the sensitivity to finding a resource. The state of the motivation level depends on the number of rewards received but not on the among-patch resource distribution: humans use a motivational mechanism of incremental arousals as a response to finding rewarding items, irrespective of the resource distribution. In the analysis of the course of motivation, we noticed that the consideration of the number of empty chests found improved the prediction, especially when the motivation drops just before leaving the patch. This effect, however, is weaker than the incremental effect of opening a filled chest.

Another variable can be used to explain the residence time. As Hutchinson *et al.* [3] found, the time since the last capture influences the decision to leave the patch. However, in their experiment, the time since the last capture was sufficient to explain the residence time. In the present work, we need to incorporate the effect of finding of an item. One possible explanation could be that humans strongly respond to the type of foraging task. The game used by Hutchinson *et al.* simulates angling, an activity in which the subject experiences a sort of ambush predation: he stays in the same place and waits until a fish takes the bait. In our game, the subject moves between the domes and between the chests inside the domes. In the first case, the time since the last discovery could be perceived as being longer by a subject who is passively foraging than in the second case, where the time between two successive discoveries is the time it takes to open the chests. It is then plausible that the evaluation of the remaining number of prey in the patch could be linked more so to a time cue in the first case than in the second one. Regardless, it is remarkable that in both experiments the human subjects seem unable to switch from the incremental rule when they face an even distribution. This is a strong convergence between two studies designed with a different interface and for a different purpose.

The experiment is based on the ability of people to communicate their motivation to stay in a patch. To accurately record the motivation course, the level of motivation felt is asked every time a chest is opened. This method could partly influence the declared motivation. Indeed, there is a strong tendency for humans to make up plausible stories to justify decisions that are actually normally determined by processes of which humans are not consciously aware [22]. Having to declare the motivation every time a chest is opened could also influence the leaving-behavior. A high declared motivation could prompt the subject to stay in the dome even if he wanted to leave. Nevertheless, the subjects were aware that the motivation level they declared had no influence at all on the score. The accuracy of the declared motivation could also be questionable. The subjective perception of a physical intensity law suggests that an exponential variation of a physical intensity of a stimulus (for example, noise) is often perceived as a linear variation. This could be applied to the proprioceptive appreciation of motivation. It is plausible that the linear mental scale for exponential variation could be responsible for the linearization of the motivational feeling. The neural basis of this phenomenon in now known and has been demonstrated in primates [38], [39]. It also could be accentuated by the experimental discretization and truncation of the motivation scale. The arbitrary scale of motivation from 0 to 9 could influence the perception of motivation and suggests a linear scale to the subject. Moreover, in many cases, when many rewards were found, the subjects gave a long series of “9” marks, when actually his real motivation level was unknown. However, the relationship between what the players declared and the statistical analysis of the patch-leaving tendency demonstrates the consistency between behavior and the declared feeling. In this sense, reported motivation is consistent with the leaving behavior observed.

Some neuroscientific studies have suggested that the motivational mechanism discovered in our study could have a neural basis in the human brain. For example, Aston-Jones and Cohen [40] demonstrated that the locus coeruleus-norepinephrine (LC-NE) system is implicated in the control of the decision to persist in a given action or switch to another. They described two neural activity patterns (phasic and tonic modes), which are similar to the proximate mechanism we found here: in the phasic mode (associated with a high level of task performance), neurons exhibit a phasic activation responding to the task-relevant stimuli. In contrast, cells fail to respond to the task-relevant stimuli during the tonic mode (associated with a poor level of task performance) [41]–[43]. Thus, the neural activity of the LC-NE system could provide a biological basis for the motivational mechanism highlighted here: during a high performance task (visiting a “good” patch), the motivation suddenly increases in response to the rewards (phasic mode of the LC-NE system). When the task performance is low (visiting a “poor” patch), the motivation decreases and the subject becomes less sensitive to the task-relevant stimuli (tonic mode).

Our study confirms that humans seem unable to adjust their response to the spatial distribution of resources because they use an incremental mechanism irrespective of the resource distribution [3], [31]. Both our study and other studies suggest that humans are adapted to finding resources with a clumped distribution over patches. Wilke and Barrett [44] expected the cognitive skills of humans to be adapted to the types of fitness-relevant problems that people faced in ancestral environments. Hunter-gatherer societies prevailed during two million years of human history. We thus hypothesize that natural selection tailored a proximate mechanism for patch leaving, which is strongly adaptive in an environment where food is distributed in an aggregated way, as is the case in hunter-gathering populations. By moving to the savanna, hominids faced dispersed but sometimes profitable food sources [45] that corresponded to an aggregative distribution of resources. In the patchy savanna environment, selection would have favored a foraging strategy that is efficient for an aggregative resource distribution. Thus, the motivational mechanism observed here and the neural mechanism detailed above are most likely adaptations that were selected for during ancestral times and are, still adaptive now for foraging on the internet or in a supermarket.

### Ethics Statement

We obtained an ethics approval for each subject by a written consent form as recommended by the French National Committee for Scientific Research (CNRS). The approval by the ethics committee was not requested by the CNRS in this case since personal nominative data were not collected.
